# AI-Assisted Speaking Practice and Human-Directed Communicative Readiness: Survey and Scenario Evidence on Anxiety, Self-Efficacy, and Evaluation Pressure

**DOI:** 10.3390/bs16101770

**Published:** 2026-09-28

**Authors:** Ruyi Wang, Jiangyu Li

**Affiliations:** 1 Faculty of Humanities and Social Sciences, University of Nottingham Ningbo China, Ningbo 315100, China; wruyiii@163.com; 2 School of Public Administration, Sichuan University, Chengdu 610065, China

**Keywords:** AI-assisted speaking practice, human-directed willingness to communicate, L2 speaking, foreign-language speaking anxiety, speaking self-efficacy, evaluation pressure

## Abstract

Generative artificial intelligence (GenAI) voice tools create low-risk opportunities for second-language (L2) speaking practice, but it remains unclear whether AI-assisted speaking practice is associated with human-directed communicative readiness and how this relationship varies across learners and evaluation contexts. Study 1 analysed cross-sectional survey data from 708 English learners. Greater AI-assisted spoken English behaviour (AISB) was associated with higher human-directed willingness to communicate (HWTC; β = 0.384) and lower foreign-language speaking anxiety (FLSA; β = −0.416); FLSA was negatively associated with HWTC when AISB was controlled (β = −0.325), yielding a positive statistical indirect association (0.127, 95% CI [0.079, 0.184]). Speaking self-efficacy (SSE) moderated the FLSA–HWTC association, including an unexpected upper-tail crossover. Demographic adjustment left the substantive pattern essentially unchanged, and an unmeasured latent method factor (ULMC) sensitivity analysis showed materially stable focal factor-score associations after accounting for a general method factor. Study 2 used a 2 × 2 nonrandom scenario comparison (N = 726), in which participants imagined preparation with an AI voice assistant versus smartphone recording under formal evaluation versus no evaluation. AI-voice-assistant scenarios showed lower anticipated FLSA and higher anticipated HWTC, whereas formal evaluation showed the opposite pattern; tool × evaluation interactions were significant for FLSA (ηp^2^ = 0.032) and HWTC (ηp^2^ = 0.047). Speaking-initiation tendency showed a different interaction pattern, indicating that HWTC and speaking-initiation tendency are not identical. The findings support a competence–affect–context account of AI-assisted speaking practice and human-directed communicative readiness, but they do not establish AI-specific causal effects or transfer to actual human speaking.

## 1. Introduction

As generative artificial intelligence expands from text generation to real-time voice interaction, L2 learners can practise speaking with few constraints related to time, location, or partner availability. Recent systematic reviews and meta-syntheses indicate that voice-based AI chatbots, speech-recognition chatbots, and conversational GenAI have become a growing area of technology-enhanced language learning. Their major affordances include high accessibility, repeated practice, immediate responses, multimodal input, personalised scaffolding, and a comparatively low-risk communication environment (Annamalai et al., 2023; Caldarini et al., 2022; J. Du & Daniel, 2024; Y. Du & Reynolds, 2025; Huang et al., 2022; Jeon et al., 2023, 2024; Jia et al., 2023; Koç & Savaş, 2025; Kohnke et al., 2023; Lai & Lee, 2024; Zhai & Wibowo, 2022). At the same time, GenAI has not made interaction, task design, or teacher mediation irrelevant. Instead, the research focus has shifted from whether AI can be used to what kinds of interactional designs can support genuine language development (Chapelle, 2024; Davin, 2024; González-Lloret, 2024; Handley, 2024; Kern, 2024; Thorne, 2024). A recent Annual Review of Applied Linguistics review of AI-supported L2 listening and speaking likewise notes that GenAI is reshaping the technological boundaries of speaking practice and assessment, while complex interpersonal interaction, authentic speaking ability, and the extent to which AI support transfers to performance beyond AI remain central questions (Goh & Aryadoust, 2025).

More direct empirical research has documented the potential value of AI-mediated speaking interaction across different technological forms. Earlier studies of voice assistants, intelligent personal assistants, and task-oriented chatbots found that learners could use Alexa, Google Assistant, speech-recognition chatbots, and task-based dialogue systems to increase speaking turns, learner autonomy, and opportunities for interaction (H. H.-J. Chen et al., 2023; Dizon, 2017, 2020; Dizon & Tang, 2020; Hsu et al., 2023; Kim et al., 2022; Moussalli & Cardoso, 2020; Yang et al., 2024). In the GenAI era, several studies in System further show that AI-mediated interaction can be associated with better EFL speaking performance and WTC and with lower FLSA (Fathi et al., 2024; C. Zhang et al., 2024). Recent studies in Computer-Assisted Language Learning, ReCALL, and TESOL Quarterly report similar patterns: embedding AI chatbots in speaking tasks may reduce anxiety, improve performance, or broaden interactional participation (Sok & Shin, 2025; Tai & Chen, 2025; T.-T. Wu et al., 2025, 2026). Research published in 2026 continues to indicate that ChatGPT voice interaction, AI chatbots, and speaking practice that combines corpora with GenAI can improve oral proficiency, WTC, positive affect, or specific speaking features (H. C. R. Chen et al., 2026; Shadiev et al., 2026; Tai, 2026; W. Zhang, 2026). Therefore, AI speaking technologies matter for both feedback provision and the creation of frequent, controllable, and psychologically manageable speaking opportunities.

From a student learning engagement perspective, AI-assisted speaking practice is a technology-supported learning activity, whereas participation in human L2 communication is a distinct downstream target. Student engagement is commonly conceptualised as behavioural, emotional, and cognitive (Fredricks et al., 2004), and the present studies do not claim to measure that full construct. Instead, drawing on MacIntyre et al.’s (1998) L2 WTC model, HWTC is treated as a proximal indicator of readiness for behavioural engagement because willingness is positioned immediately before communicative action. In this article, AI-assisted spoken English behaviour (AISB) refers specifically to the frequency and intensity of learners’ actual voice-based English interaction with AI during the preceding four weeks, whereas human-directed willingness to communicate (HWTC) refers specifically to readiness to speak with human teachers, peers, and less-familiar human interlocutors rather than with AI. FLSA is treated as an affective barrier, SSE as a competence belief and individual-difference condition, and evaluation pressure as an educational contextual condition. This mapping can be used to make this transition from AI-assisted speaking practice to human-directed communicative readiness explicit but not to equate readiness with observed participation, or broader engagement, or learning transfer.

Two related gaps motivate the studies. First, it is often reported in speaking research with the support of AI that there is WTC, anxiety, or oral performance after the use of technology, but the target of communication is not clearly distinguished. Recent work has begun to examine willingness to communicate with AI as a construct in its own right, reinforcing the need to separate AI-directed WTC from human-directed WTC (Peng & Liang, 2025; Zou et al., 2025). WTC is inherently situated: interlocutor type, classroom environment, group size, momentary emotion, and perceived competence can all affect whether a learner speaks (Cao, 2011; Freiermuth & Jarrell, 2006; Khajavy et al., 2018; C. Li et al., 2025; Z. Li et al., 2025; MacIntyre et al., 1998; MacIntyre, 2007; Peng & Woodrow, 2010; Yashima, 2002). Successful interaction with AI therefore cannot be assumed to imply readiness for a human classroom, still less demonstrated transfer. Second, although AI-assisted speaking practice is often described as low-threat, learners eventually return to discussions, presentations, and assessments involving teacher grading, peer comparison, and possible negative evaluation. Speaking anxiety can vary with medium, visibility, communication mode, and evaluative risk (Bárkányi & Brash, 2025; Topalov et al., 2023; Tutton & Cohen, 2024; Woodrow, 2006).

Accordingly, the broad question of whether AI-assisted speaking practice is effective is reframed as a question about when AI-assisted speaking practice is associated with human-directed communicative readiness. Study 1 asks whether AISB is associated with HWTC through FLSA and whether SSE conditions that association. Study 2 asks whether anticipated FLSA and HWTC differ across controlled preparation-tool and evaluation-pressure scenarios for an upcoming human discussion. The two designs are complementary rather than interchangeable: Study 1 captures recent AISB but is cross-sectional and associational; Study 2 standardises the imagined preparation and evaluation context but measures anticipated responses rather than behaviour after completed practice. Having both designs allows for triangulation between naturalistic association and controlled scenario appraisal while maintaining the evidential boundaries of each. Both studies do not directly examine realised behavioural involvement in actual class.

## 2. Theoretical Framework and Hypotheses

### 2.1. AI Speaking Practice, Foreign-Language Speaking Anxiety, and Human-Directed Willingness to Communicate

MacIntyre et al.’s (1998) L2 WTC model places willingness to communicate immediately before actual language use and identifies perceived communicative competence and situational anxiety as proximal antecedents of communicative action. Self-efficacy theory adds a competence-appraisal mechanism: learners who judge themselves capable of managing a speaking task are more likely to persist and regulate affect adaptively (Bandura, 1997; Mills et al., 2007). Evaluative-anxiety perspectives further emphasise fear of errors, social exposure, and negative evaluation as contextual sources of speaking threat (Horwitz et al., 1986; Woodrow, 2006). Integrating these perspectives, the present framework treats AI-assisted speaking practice as a preparation context that may relate to human-directed communicative readiness partly through FLSA; SSE conditions how anxiety is translated into readiness, while evaluation pressure changes the social-evaluative context in which readiness is anticipated. This competence–affect–context synthesis therefore distinguishes the activity, affective mechanism, competence belief, and contextual boundary rather than treating AI-assisted speaking practice as a unitary cause of communication.

AI voice assistants provide a conversational environment that differs from a human interlocutor: learners can repeat utterances, request clarification, receive immediate responses, and often practise without direct human judgement. Research on chatbots, intelligent personal assistants, and GenAI speaking tools has linked these affordances to increased speaking opportunities, autonomy, self-efficacy, lower anxiety, higher WTC, and, in some studies, better speaking performance (Çakmak, 2022; El Shazly, 2021; Fathi et al., 2024; Fryer et al., 2017, 2019, 2020; Hwang et al., 2024; S. Lee & Jeon, 2024; C. Wang et al., 2024; T.-T. Wu et al., 2025; C. Zhang et al., 2024; W. Zhang, 2026). The proposed pathway is therefore AISB → lower FLSA → higher HWTC, alongside a direct AISB–HWTC association that may reflect familiarity, rehearsal, or other unmeasured aspects of repeated speaking activity. Because Study 1 is cross-sectional, these arrows represent hypothesised statistical associations rather than temporal causal effects.

Speaking self-efficacy (SSE) refers to learners’ judgements that they can successfully perform specific speaking tasks. Higher SSE is generally associated with greater persistence, more adaptive emotion regulation, and stronger readiness to communicate (Bandura, 1997; Mills et al., 2007; Peng & Woodrow, 2010). In technology-supported speaking environments, successful interaction and repeated practice may also inform competence beliefs (Bárkányi, 2021; Fryer et al., 2020; X. Wang et al., 2023; C. Wang et al., 2024). Within the present model, SSE is therefore a boundary condition on the FLSA–HWTC link: the primary expectation is that strong competence beliefs buffer the negative association between anxiety and readiness to communicate.

An alternative pattern is also theoretically possible. From an emotional-arousal perspective, learners may interpret the same nervous arousal differently depending on competence beliefs. Lower-SSE learners may read arousal as evidence of insufficient ability, error exposure, or social threat, whereas some higher-SSE learners may experience moderate arousal as alertness or task activation rather than a signal to avoid communication. The a priori prediction therefore remains attenuation of the negative FLSA–HWTC association as SSE rises; a flattening or crossover would instead be treated as a counter-intuitive boundary pattern requiring replication, not as evidence that speaking anxiety is generally beneficial. This distinction makes the expected and plausible alternative patterns explicit before hypothesis testing.

Accordingly, Study 1 proposed the following hypotheses:

**H1.**  *AI-assisted spoken English behaviour (AISB) is positively associated with human-directed willingness to communicate (HWTC);*

**H2a.**  *AISB is negatively associated with foreign-language speaking anxiety (FLSA);*

**H2b.**  *FLSA is negatively associated with HWTC;*

**H2c.**  *AISB has a positive indirect association with HWTC through lower FLSA;*

**H3.**  *SSE moderates the relationship between FLSA and HWTC such that the negative association of FLSA becomes weaker as SSE increases;*

**H4.**  *The positive indirect association between AISB and HWTC through FLSA becomes weaker as SSE increases.*

**Figure 1:**
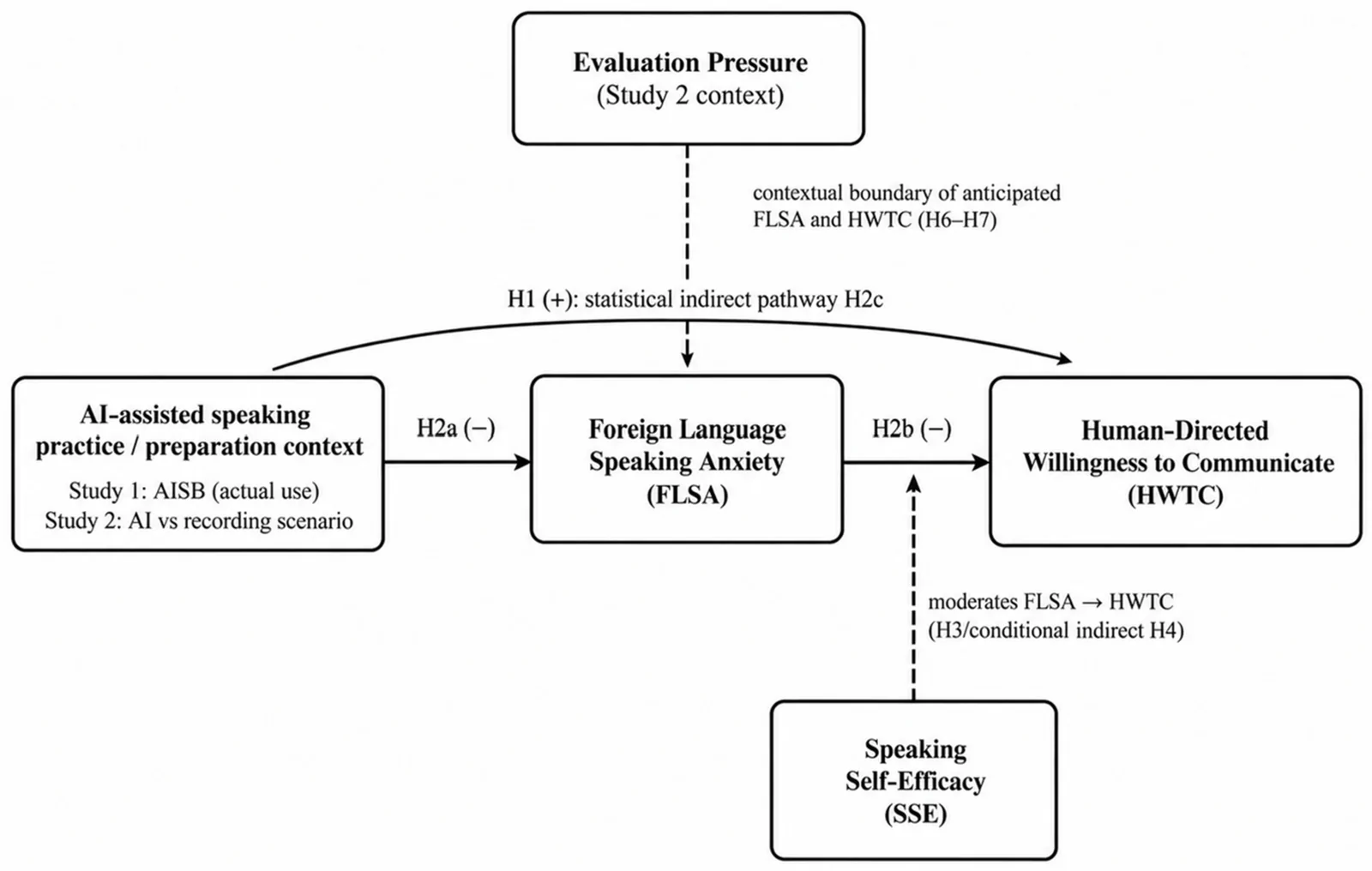
Integrative competence–affect–context framework across Studies 1 and 2. Study 1 examines AISB → FLSA → HWTC with SSE moderating the FLSA–HWTC association; Study 2 examines preparation-tool and evaluation-pressure scenario differences in anticipated FLSA and HWTC. Evaluation pressure is a contextual factor in Study 2 rather than a variable measured in Study 1.

The integrative framework across the two studies is summarised in Figure 1. Study 1 tests the AISB → FLSA → HWTC pathway and SSE moderation, whereas Study 2 probes preparation-tool and evaluation-pressure differences in anticipated FLSA and HWTC.

### 2.2. Evaluation Pressure as a Contextual Boundary of Human-Directed Communicative Readiness

If a central affordance of AI-assisted speaking practice is reduced social-evaluative threat, the evaluation structure that learners expect when they return to human communication becomes an important contextual boundary. Foreign-language anxiety theory identifies test anxiety and fear of negative evaluation as central sources of classroom threat (Horwitz et al., 1986), and speaking-anxiety research shows that observation by teachers or peers, face-to-face exposure, and concern about language errors can heighten anxiety (Botes et al., 2020; Woodrow, 2006). Online-learning research likewise shows that participation changes with cameras, recording, communication medium, self-disclosure, and perceived risk of negative evaluation (Bárkányi & Brash, 2025; Topalov et al., 2023; Tutton & Cohen, 2024). The WTC framework and classroom-environment research therefore converge on the expectation that formal grading should increase anticipated FLSA and reduce anticipated HWTC even when preparation time and task content are held constant.

An AI voice assistant also differs from smartphone recording in dialogicality: it can respond, ask follow-up questions and provide feedback, whereas recording is essentially a monologic record–replay activity. Prior research suggests that task design, dialogue coherence, social presence, embodiment, feedback, and interaction mode can shape AI-supported speaking outcomes (Ebadi & Amini, 2024; Ericsson et al., 2023, 2024; Han & Lee, 2024; Jeon, 2024; K.-A. Lee & Lim, 2023; C. H. Wu et al., 2023; Xiao et al., 2024; Yuan, 2024). Accordingly, the expected main-effect pattern is lower anticipated FLSA and higher anticipated HWTC for the AI-voice-assistant scenario. A compensatory interaction is also plausible: if interactive rehearsal is perceived as especially useful when social-evaluative threat is high, the AI–recording difference should widen under evaluation. An additive or null interaction remains a theoretically meaningful alternative if preparation-tool affordances and evaluation pressure operate largely independently. Importantly, because the two tool scenarios differ simultaneously in interactivity, responsiveness, and feedback, Study 2 cannot isolate an AI identity effect from these bundled dialogic affordances.

Accordingly, Study 2 proposed the following hypotheses:

**H5.**  *In the preparation-tool scenarios, FLSA is lower and HWTC is higher in the AI-voice-assistant condition than in the smartphone recording condition;*

**H6.**  *In the evaluation-pressure scenarios, FLSA is higher and HWTC is lower under formal evaluation than under no evaluation;*

**H7.**  *Preparation tool and evaluation pressure interact statistically, such that the between-scenario difference between the AI-voice-assistant and smartphone-recording conditions is larger under evaluation pressure.*

## 3. Study 1: Survey Study of AI-Assisted Spoken English Behaviour and Human-Directed Willingness to Communicate

### 3.1. Participants and Data Collection

Study 1 used a one-time cross-sectional online survey with 708 valid cases. All participants were aged 18 years or older, were learning or using English, and reported their AISB based on their actual experience during the preceding four weeks. The sample included 157 men (22.2%) and 551 women (77.8%); 363 participants were undergraduates (51.3%), 196 were diploma/vocational-college students (27.7%), 121 were master’s students (17.1%), and 28 were doctoral students (4.0%). The questionnaire was administered anonymously in Chinese and English. The opening page explained that participation was voluntary, that respondents could withdraw before submission, and that they were required to provide explicit consent before proceeding.

### 3.2. Measures

AI-assisted spoken English behaviour (AISB) was operationalised as an observed behavioural composite based on three ordered indicators covering the preceding four weeks, namely number of interaction sessions, cumulative interaction time, and number of days of use. We excluded text-only input and listening to AI-generated speech. Because the three indicators capture distinct aspects of use intensity rather than interchangeable manifestations of a reflective construct, AISB is treated as a composite index, and Cronbach’s alpha is not used as its primary measurement criterion. FLSA, SSE, and HWTC each comprised five contextualised items, with α = 0.847, 0.834, and 0.833, respectively. A three-factor measurement model showed good fit (χ^2^(87) = 115.31, χ^2^/df = 1.325, CFI = 0.993, TLI = 0.992, RMSEA = 0.021, SRMR = 0.024). Standardised item loadings ranged from 0.680 to 0.743, composite reliability (CR) ranged from 0.833 to 0.847, and AVE ranged from 0.499 to 0.526. HTMT values ranged from 0.485 to 0.511, and the Fornell–Larcker criterion was met. Because HWTC AVE was 0.499, essentially at the conventional 0.50 benchmark, its convergent-validity evidence is interpreted as borderline rather than unequivocal. Full item-level evidence is reported in Table 1.

AISB scoring was based on the three use-intensity indicators only. For each indicator, the six ordered response categories were coded 0–5. Session frequency was coded as 0, 1–2, 3–5, 6–9, 10–19, and ≥20 sessions; cumulative time as 0, 1–15, 16–30, 31–60, 61–120, and ≥121 min; and use days as 0, 1–3, 4–7, 8–12, 13–20, and 21–28 days. The three equally weighted codes were then averaged: AISB = (session code + cumulative-time code + use-days code)/3. The separate yes/no AI-use item was used for consistency checking and was not included in this mean index.

The five FLSA items were selected and contextually adapted from the L2 speaking-anxiety construct in Woodrow (2006). The five SSE items were developed with reference to the English-speaking self-efficacy frameworks of Gan et al. (2022) and Y. Wang and Sun (2024), using can-do capability judgements. The five HWTC items were selected and contextually adapted from human communication situations in J. S. Lee et al. (2021). These are contextualised short forms rather than unmodified reproductions of the source instruments. The exact administered English wording, response anchors, and scoring rules are reported in Appendix A. The study did not include an independent expert-panel content-validity index or a separate pilot study specifically validating these shortened adaptations. Construct validity was therefore evaluated through item loadings, CR, AVE, HTMT, and Fornell–Larcker evidence, while the absence of independent content-validity testing is treated as a limitation for future validation work.

### 3.3. Measurement Quality and Analytical Strategy

The analyses proceeded through descriptive statistics and correlations, simple regression, bootstrap estimation of statistical indirect associations, FLSA × SSE moderation, conditional indirect associations, Johnson–Neyman probing, HC3 heteroskedasticity-robust estimation, and a quadratic specification. Descriptive statistics and correlations for the core Study 1 variables are reported in Table 2. Statistical indirect and conditional indirect associations were estimated using 5000 bootstrap resamples with 95% confidence intervals. Continuous variables were centred before the interaction term was constructed, and conditional associations were examined at the SSE mean and one standard deviation below and above the mean. A statistical indirect or conditional indirect association was considered statistically significant when its bootstrap confidence interval did not include zero. Common method bias (CMB) was addressed procedurally through anonymous responses, separation of the FLSA, SSE, and HWTC sections, and different response anchors for agreement, capability judgements, and willingness. The first factor in the unrotated Harman analysis accounted for 34.615% of variance, which only indicates that a single dominant factor was not evident and does not establish the absence of CMB.

An unmeasured latent method factor (ULMC) sensitivity model was also estimated by adding one orthogonal method factor loading on all 15 FLSA, SSE, and HWTC items. The model retained acceptable fit (χ^2^(72) = 71.00, CFI = 1.000, TLI = 1.000, RMSEA = 0.000, SRMR = 0.021). Method loadings were mixed in sign and modest in magnitude (range = −0.265 to 0.212; mean absolute loading = 0.099). Factor-score estimates were materially stable after accounting for the method factor: AISB→FLSA changed from −0.429 to −0.428, the FLSA→HWTC association controlling AISB from −0.496 to −0.491, and the corresponding statistical indirect estimate from 0.213 to 0.210. These results reduce concern that one general method factor drives the focal pattern, but they do not eliminate CMB because Study 1 remains a single-wave self-report survey without temporal separation, independent data sources, or a marker variable.

### 3.4. Hypothesis Testing

AISB was significantly and positively associated with HWTC, B = 0.359, SE = 0.033, β = 0.384, t = 11.058, *p* < 0.001, R^2^ = 0.148, supporting the associational prediction in H1. AISB was significantly and negatively associated with FLSA, B = −0.404, β = −0.416, *p* < 0.001, supporting H2a. When AISB was included simultaneously, FLSA remained significantly and negatively associated with HWTC, B = −0.313, β = −0.325, *p* < 0.001, supporting H2b. In the cross-sectional conditional-process model, the estimated statistical indirect association between AISB and HWTC through FLSA was 0.127, 95% CI [0.079, 0.184], supporting the indirect-association prediction in H2c. This estimate should not be interpreted as demonstrating that AISB first caused changes in anxiety that subsequently caused changes in HWTC.

The FLSA × SSE interaction was significant, B = 0.281, *p* < 0.001. Simple-slope results showed a relatively strong negative relationship between FLSA and HWTC at low SSE (slope = −0.371), whereas the fitted slope became positive at high SSE (+0.137). The index of moderated indirect association was −0.109, 95% CI [−0.145, −0.075]. The conditional indirect association declined from 0.142 at low SSE to 0.044 at mean SSE and reversed to −0.054 at high SSE. Thus, SSE significantly altered both the statistical FLSA–HWTC relationship and the conditional indirect association, but the functional form differed from the a priori expectation of monotonic attenuation. H3 and H4 were therefore only partially supported. Although the moderation effect reached statistical significance, the effect direction observed under high-level SSE did not align with our initial hypotheses. Instead of treating this crossover result as evidence that anxiety improves communication, we conducted further follow-up analyses to unpack this effect. The main path estimates and conditional indirect associations are summarised in Table 3.

### 3.5. Robustness Analyses

Additional probing analyses for the crossover effect were performed. Two transition points on the observed SSE scale were detected via Johnson–Neyman analysis, at approximately 3.88 and 4.45. The conditional association between FLSA and HWTC was significantly negative for values below SSE ≈ 3.88, non-significant across the SSE range of 3.88 to 4.45, and significantly positive above SSE ≈ 4.45. Nearly identical threshold bounds (roughly 3.87 and 4.45) were obtained when HC3 heteroskedasticity-robust standard errors were applied, and the FLSA × SSE interaction was still statistically significant (B = 0.281, SE = 0.022, *p* < 0.001). A quadratic model incorporating FLSA^2^ and SSE^2^ was also tested. The interaction remained significant (B = 0.269, *p* < 0.001), whereas neither quadratic term reached statistical significance (*p* = 0.497 and *p* = 0.364, respectively). Given that only 55 participants (7.8%) were located above the upper Johnson–Neyman threshold on the SSE scale, the positive slope at high SSE values should be interpreted with caution. This upper-tail crossover pattern is therefore emphasised rather than taken as empirical evidence for facilitative speaking anxiety.

A demographic-adjusted analysis entered gender and education level as covariates in the Study 1 regression, indirect-association, and moderation models. The focal estimates were essentially unchanged: AISB remained positively associated with HWTC (B = 0.357, β = 0.382, *p* < 0.001) and negatively associated with FLSA (B = −0.404, β = −0.416, *p* < 0.001); FLSA remained negatively associated with HWTC when AISB and demographics were controlled (B = −0.312, β = −0.324, *p* < 0.001). The adjusted statistical indirect association was 0.126, 95% CI [0.078, 0.182]. The FLSA × SSE interaction also remained significant (B = 0.271, *p* < 0.001), with an index of moderated indirect association of −0.109, 95% CI [−0.146, −0.076]. Conditional indirect estimates were 0.141 at low SSE, 0.043 at mean SSE, and −0.056 at high SSE. Gender and education coefficients were not statistically significant in the focal Study 1 models (all *p* > 0.05). Thus, the core associations were robust to demographic adjustment.

## 4. Study 2: Scenario-Based Study of AI Voice Assistant Preparation and Evaluation Pressure

Study 2 investigated whether there were any differences between anticipated FLSA and HWTC, based on the standardised hypothetical preparation and evaluation contexts, extending the results of Study 1. Study 1 captured naturalistic AISB, but it could not isolate the effects of preparation context and evaluation pressure. Study 2 therefore used a 2 × 2 scenario-based design to compare two different modes of preparation (preparation with an AI voice assistant versus smartphone recording) with the two evaluation conditions (formal evaluation versus no evaluation) for the forthcoming human group discussion. The aim was not to show how AI-assisted speaking practice affects real-life speaking behaviour but to compare the expected appraisals of the two scenario contrasts.

### 4.1. Scenario Development and Design

Study 2 used a 2 (preparation tool: AI voice assistant/smartphone recording) × 2 (evaluation pressure: present/absent) between-subjects scenario-based group design. Participants read and imagined that they would take part the following week in an approximately five-minute English discussion with two other students after ten minutes of preparation. In the AI-voice-assistant scenario, the voice assistant was described as listening, responding in English, asking follow-up questions, and giving brief feedback on vocabulary, grammar, or clarity. In the smartphone-recording scenario, the phone could only record, replay, and re-record speech and could not respond or provide language feedback. The evaluation condition stated that individual speaking performance would be formally assessed and contribute to the course grade; the no-evaluation condition described regular ungraded practice. Other task information was held constant. This preparation-tool contrast deliberately distinguishes an interactive dialogic tool from a monologic record–replay benchmark, but it therefore bundles AI identity with responsiveness, dialogue, and feedback. The design cannot determine whether any difference is uniquely attributable to AI rather than to those dialogic affordances. A human dialogic partner or feedback-capable virtual avatar was not included, which is a key comparison for future real-task research. FLSA and HWTC are therefore anticipated responses to potential scenarios, not post-intervention outcomes.

### 4.2. Participants and Procedure

Data were gathered via four distinct survey links, each corresponding to one experimental condition. Participants were not allocated to conditions by a unified system upon study entry. For this reason, strong causal terminology, including randomised experiment, random assignment, and treatment effect, is avoided within the manuscript. Of 730 initial responses, 726 valid cases were retained after applying informed consent, age, student status, and minimum completion-time (45 s) criteria: 167 in Group A (AI × evaluation), 188 in Group B (AI × no evaluation), 184 in Group C (recording × evaluation), and 187 in Group D (recording × no evaluation). The sample included 302 men (41.6%) and 424 women (58.4%), and mean completion time was 84.6 s (SD = 13.3). Gender, stage of study, and disciplinary category did not differ significantly across the four groups, nor were the main effects of preparation tool or evaluation pressure, or their interaction, significant for pre-scenario SSE.

### 4.3. Measures, Manipulation Checks, and Descriptive Statistics

The primary outcomes were scenario-specific FLSA (five items, α = 0.865) and scenario-specific HWTC (five items, α = 0.842); pre-scenario SSE was measured with five items (α = 0.795). Speaking-initiation tendency is reverse-scored from preferred speaking order and, throughout the manuscript, refers to an intention and not an observed speaking time to speak. There were two items measuring perceived tool interactivity (MC, α = 0.732) and two items measuring perceived evaluation pressure (EVAL, α = 0.725). Two other items measured scenario realism (α = 0.569), and participants also indicated their previous frequency of use of AI voices. The manipulation checks followed the primary outcomes to reduce direct cueing, although demand characteristics remain possible because the items were semantically close to the scenario manipulations. The two realism items were treated descriptively rather than as a latent construct or exclusion criterion. Primary analyses used 2 × 2 ANOVA with Type III sums of squares. Robustness ANCOVAs included pre-scenario SSE, prior AI voice-use frequency, gender, and education level as covariates, with education treated categorically.

The manipulation checks showed that perceived tool interactivity was higher in the AI-voice-assistant conditions (M = 3.90, SD = 0.85) than in the smartphone recording conditions (M = 2.50, SD = 0.76), t(707.2) = 23.32, *p* < 0.001, d = 1.74. Perceived evaluation pressure was also higher in the evaluation conditions (M = 3.90, SD = 0.84) than in the no-evaluation conditions (M = 2.50, SD = 0.73), t(697.3) = 23.89, *p* < 0.001, d = 1.78. These large differences indicate that participants could identify the explicit contrasts between the AI-voice-assistant versus smartphone-recording conditions and formal evaluation versus no evaluation in the scenario materials. Passing the manipulation checks, however, supports recognition of the conditions only; it does not demonstrate that the hypothesised psychological mechanisms were activated, nor does it remedy the external-validity limitations of nonrandom link-based assignment or imagined scenarios. Because the manipulation checks were self-report items within the same questionnaire and closely matched the wording of the manipulations, they may also partly reflect demand characteristics. Subsequent group differences are therefore interpreted as controlled scenario-condition comparisons.

**Figure 2:**
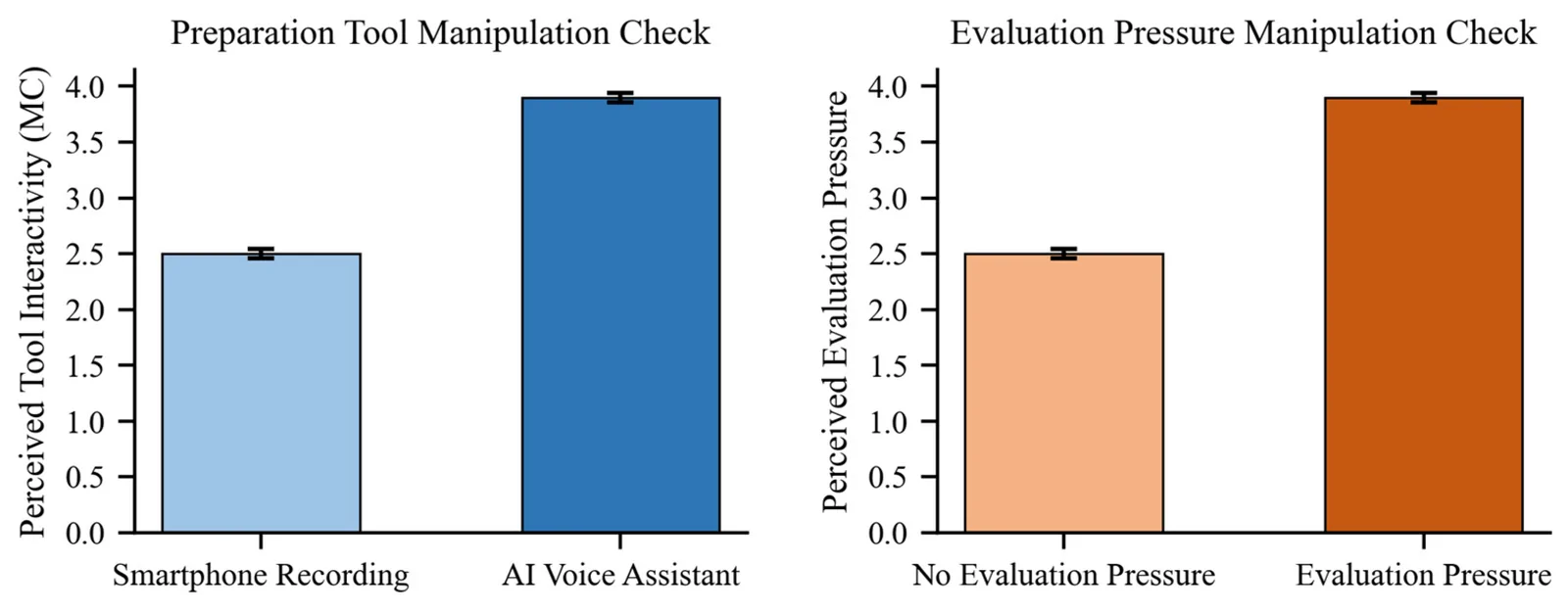
Manipulation checks for preparation tool and evaluation pressure in Study 2.

Figure 2 visualises the manipulation-check contrasts for preparation tool and evaluation pressure.

The descriptive statistics showed clear cell ordering. Group B (AI × no evaluation) had the lowest FLSA (M = 2.70) and the highest HWTC (M = 3.60), whereas Group C (recording × evaluation) had the highest FLSA (M = 4.05) and the lowest HWTC (M = 2.50). Groups A and D fell between these two conditions. Speaking-initiation tendency followed a similar descriptive pattern, with the highest mean in Group B (M = 3.86) and the lowest in Group C (M = 2.95). Mean scenario-realism scores ranged from approximately 3.15 to 3.30 across groups, suggesting that the materials were generally imaginable but not equivalent to emotions or behaviour experienced in an actual classroom. Descriptive statistics for the four Study 2 conditions are presented in Table 4.

**Figure 3:**
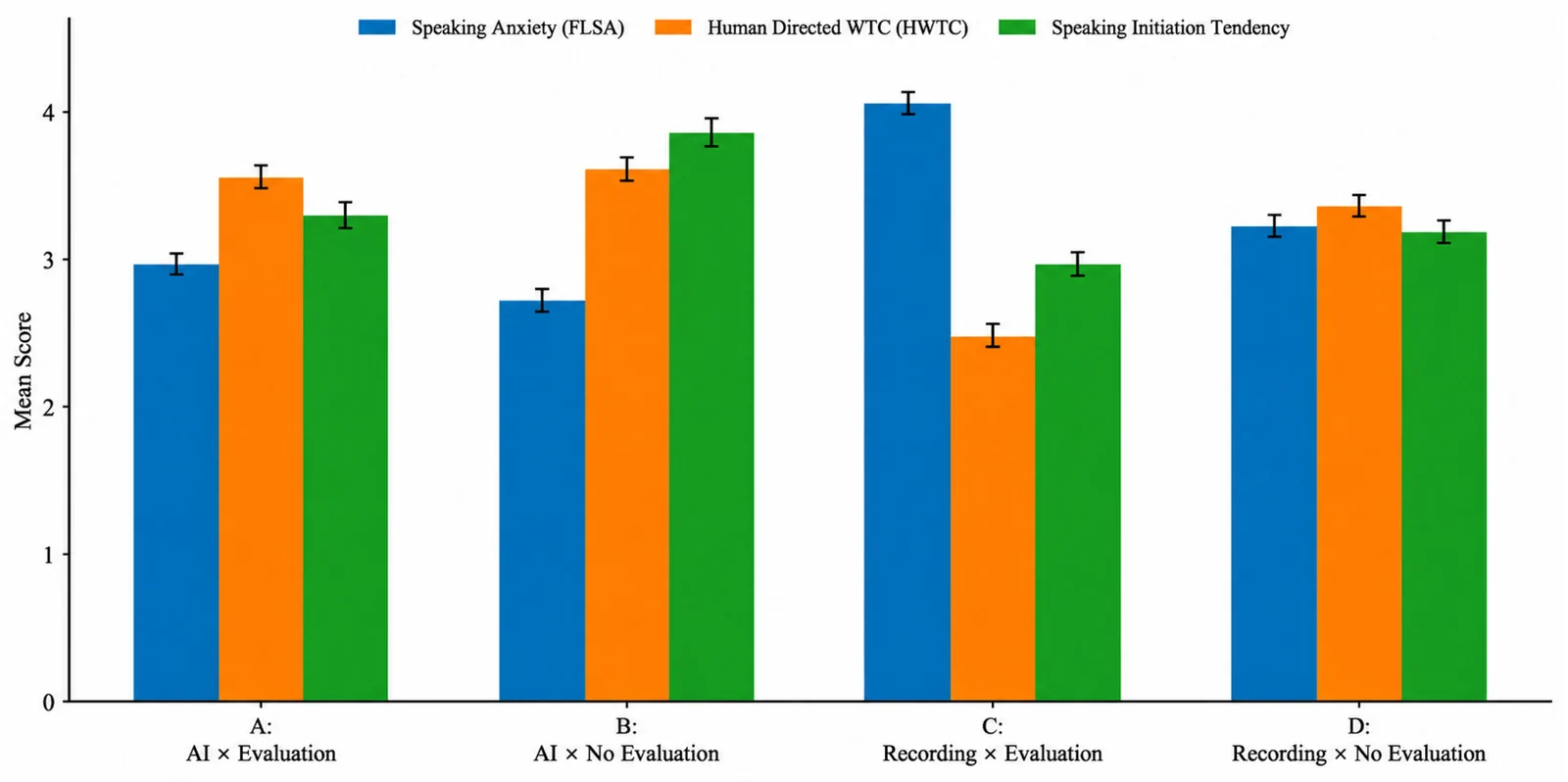
Descriptive comparison of FLSA, HWTC, and speaking-initiation tendency across the four Study 2 conditions.

A descriptive comparison of the three main outcomes across the four Study 2 conditions is shown in Figure 3.

The exact administered English wording and response anchors for pre-scenario SSE, scenario-specific FLSA and HWTC, the speaking-order item, manipulation checks, scenario-realism items, and the prior AI voice-use question are provided in Appendix A.

### 4.4. Hypothesis Testing

For FLSA, anticipated anxiety was lower in the AI-voice-assistant scenarios than in the smartphone-recording scenarios and higher under formal evaluation than under no evaluation. Both main effects and the preparation tool × evaluation pressure interaction were significant, with the largest effect associated with preparation tool. The AI–recording difference was also larger under evaluation (ΔM = −1.10) than without evaluation (ΔM = −0.50). These findings are consistent with H5–H7 at the level of scenario-condition differences. Because allocation was not individually randomised and no practice was actually delivered, the estimates are not interpreted as treatment effects or causal reductions in anxiety.

**Figure 4:**
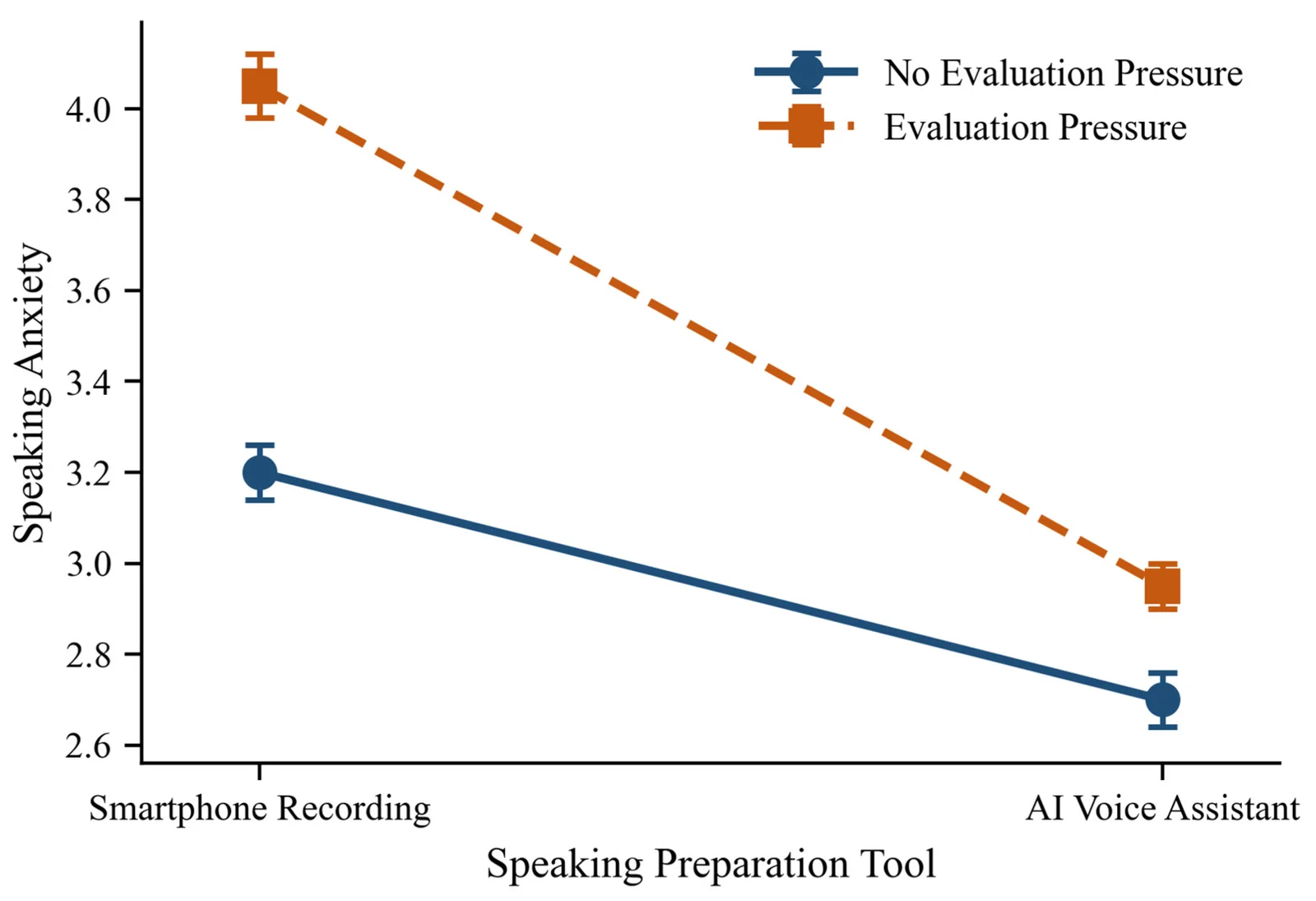
Interaction of preparation tool and evaluation pressure on anticipated FLSA in Study 2.

The FLSA interaction pattern is illustrated in Figure 4.

For HWTC, anticipated willingness was higher in the AI-voice-assistant scenarios and higher without formal evaluation. The two main effects and their interaction were significant. The AI–recording difference was substantially larger under evaluation (ΔM = 1.05, d = 1.35) than without evaluation (ΔM = 0.30, d = 0.34). The pattern therefore supports H5–H7 as a scenario-condition comparison while remaining noncausal because the conditions were not individually randomised and participants did not complete the preparation activities.

**Figure 5:**
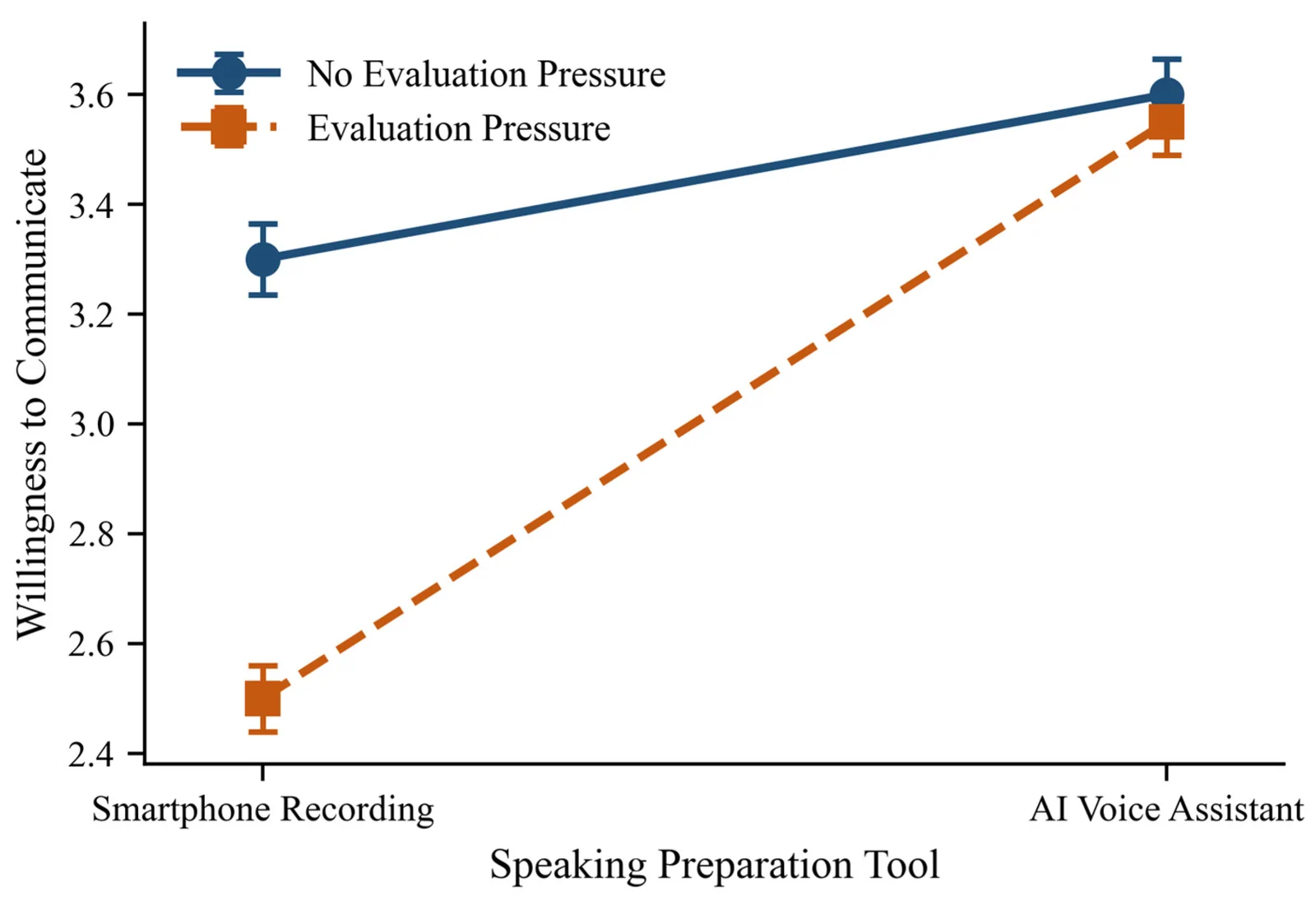
Interaction of preparation tool and evaluation pressure on anticipated HWTC in Study 2.

The corresponding interaction for HWTC is shown in Figure 5.

Speaking-initiation tendency, treated as a secondary behavioural-intention indicator, also showed significant effects of preparation tool, evaluation pressure, and their interaction. Group B had the highest speaking-initiation tendency (M = 3.86), whereas Group C had the lowest (M = 2.95). Unlike HWTC, however, the AI–recording difference was larger without evaluation pressure (d = 0.58) than under evaluation pressure (d = 0.31). This directionally different interaction suggests that willingness to take an early, publicly exposed turn is not equivalent to broader readiness to participate across communicative acts. Speaking-initiation tendency is therefore interpreted as a distinct secondary outcome rather than as another measure of HWTC.

**Figure 6:**
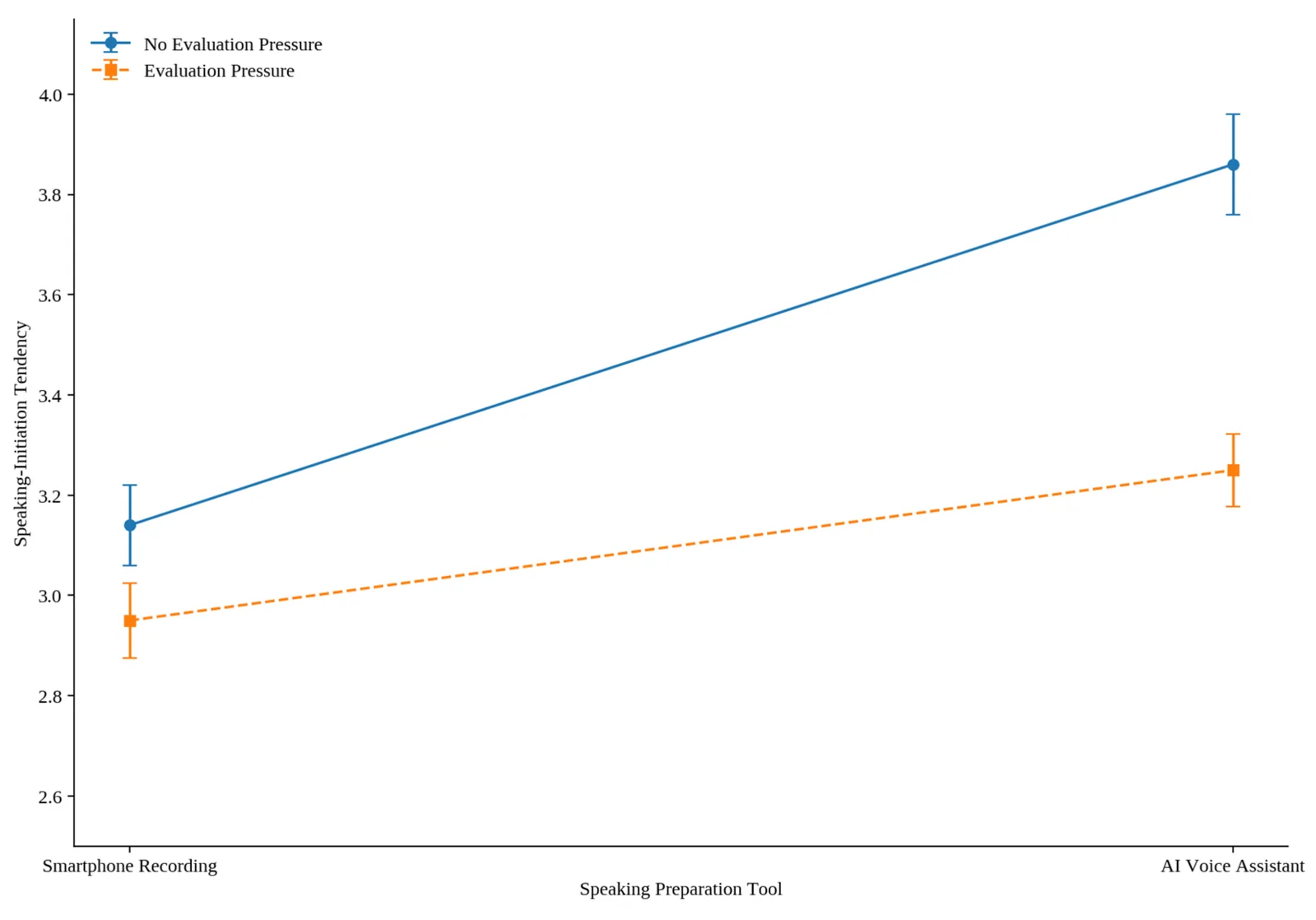
Interaction of preparation tool and evaluation pressure on speaking-initiation tendency in Study 2.

The interaction for speaking-initiation tendency is displayed in Figure 6. The primary ANOVA results and tool contrasts for all three outcomes are summarised in Table 5.

### 4.5. Robustness Analyses

After controlling simultaneously for pre-scenario SSE, prior AI voice-use frequency, gender, and education level, the core scenario-condition pattern remained stable. For FLSA, preparation tool remained significant, F(1,716) = 204.90, *p* < 0.001, ηp^2^ = 0.223; evaluation pressure remained significant, F(1,716) = 95.26, *p* < 0.001, ηp^2^ = 0.117; and the interaction remained significant, F(1,716) = 31.30, *p* < 0.001, ηp^2^ = 0.042. For HWTC, preparation tool remained significant, F(1,716) = 140.95, *p* < 0.001, ηp^2^ = 0.164; evaluation pressure remained significant, F(1,716) = 57.47, *p* < 0.001, ηp^2^ = 0.074; and the interaction remained significant, F(1,716) = 39.01, *p* < 0.001, ηp^2^ = 0.052. Education level was not significant for either outcome; gender was not significant for FLSA and showed only a small association with HWTC (ηp^2^ = 0.006). SSE remained associated with both outcomes, and prior AI-use effects were small. These analyses confirm that the focal scenario contrasts are not explained by the measured demographic covariates, but they do not recreate randomisation or rule out unmeasured selection bias. The adjusted ANCOVA results for both outcomes are summarised in Table 6.

The robustness models still did not include objective proficiency indicators such as CEFR level, IELTS/TOEFL scores, speaking-course grades, or blindly rated speech samples, nor did they control directly for recent frequency of human English interaction or prior experience with classroom evaluation. SSE is a competence belief rather than an objective proficiency proxy. Accordingly, persistence of the tool × evaluation interactions after demographic and SSE adjustment should be interpreted as statistical robustness within the observed sample, not as proof of baseline equivalence or causal effects.

## 5. Conclusions and Discussion

### 5.1. Overall Findings and Interpretation

In Study 1, greater AISB was associated with lower FLSA and higher HWTC, with a significant statistical indirect association involving FLSA. In Study 2, participants anticipating the AI-voice-assistant scenario reported lower FLSA and higher HWTC than those anticipating smartphone recording, and the between-scenario difference for the two primary outcomes was larger under formal evaluation. However, Study 2 contrasted an interactive, feedback-capable dialogic tool with a noninteractive monologic smartphone-recording tool. The resulting difference is therefore partly expected on affordance grounds and cannot be attributed uniquely to AI identity. Neither study observed human speaking behaviour after completing AI-assisted speaking practice. The evidence concerns human-directed communicative readiness and anticipated affect, not demonstrated learning transfer or an AI-specific causal treatment effect.

A major contribution of the present research is to make the communication target explicit as a part of the explanatory account. Existing studies on AI speaking mainly focused on either general WTC or speaking scores post-intervention, and recent research has started to directly explore the willingness to communicate with ChatGPT/AI and learner adoption behaviour (Peng & Liang, 2025; Zou et al., 2025). This distinction means that willingness to communicate with AI cannot automatically substitute for human-directed willingness to communicate. Study 1 explicitly anchors HWTC in human communication, and Study 2 specifies an imagined transition from AI-voice-assistant/smartphone-recording preparation to an upcoming human group discussion. This approach responds to a central concern raised by Goh and Aryadoust (2025) and Kern (2024): technology can expand speaking opportunities. However, the goal of L2 speaking remains complex interpersonal interaction. Future GenAI speaking research should therefore measure performance within AI, actual human interaction behaviour, and delayed transfer outcomes separately rather than treating readiness, behaviour, and transfer as interchangeable outcomes.

The indirect-association results in Study 1 and the controlled scenario-condition differences in Study 2 identify FLSA as an important affective correlate of human-directed communicative readiness while not establishing it as a causal bridge from AI-assisted speaking practice to later human behaviour. Recent AI speaking research repeatedly reports lower anxiety during or following AI interaction (Ballıdağ & Aydın, 2025; C. Wang et al., 2024; T.-T. Wu et al., 2025; C. Zhang et al., 2024). Research on speech-enabled corrective feedback, mobile speaking tasks, and online speaking environments likewise shows that technological design can alter anxiety and confidence (Y.-C. Chen, 2024; Ebadi & Azizimajd, 2026; Shadiev et al., 2026). Systematic reviews and meta-syntheses further identify a safe practice space and repeatable interaction as important mechanisms through which AI chatbots may support speaking (J. Du & Daniel, 2024; Huang et al., 2022; Koç & Savaş, 2025). The present findings further show that affective differences covary with HWTC.

However, the challenging finding in Study 1, that anxiety is reduced via an upper-tail crossover, needs more explanation than simply saying SSE reduces anxiety. Johnson–Neyman probing revealed that FLSA and HWTC were significantly negatively associated at lower values of SSE ≈ 3.88, non-significantly associated at intermediate values of SSE (≈3.88 to 4.45), and significantly positively associated at higher values of SSE >≈ 4.45. The HC3 estimation gave almost the same transition points and did not remove the interaction (B = 0.281, SE = 0.022, *p* < 0.001), even with the inclusion of FLSA^2^ and SSE^2^. There are three possible, but not mutually exclusive, explanations. First, high-SSE students might think some amount of activation is challenge, alertness or activation of the task, and not a signal that they are unable to cope, and so, the normal avoidance pathway is lessened. Second, if the statistical conditioning is performed on a competence belief which is positively associated with HWTC and negatively associated with FLSA, it may reveal a small upper-tail association which is not indicative of a causal benefit of anxiety. Thirdly, there were only 55 participants (7.8% of Study 1) in the positive region, leaving sampling instability, restricted range or differences in proficiency due to unmeasured factors as viable alternatives. The crossover should, therefore, be seen as a theoretically interesting but weak boundary pattern that needs to be preregistered and replicated objectively with proficiency and repeated observations and not seen as evidence that anxiety promotes communication.

The Study 2 ANOVA showed a significant main effect of evaluation pressure and a significant statistical interaction with the preparation-tool scenario. Formal evaluation was associated with higher anticipated FLSA and lower anticipated HWTC, indicating that grading and grade consequences were salient within participants’ imagined human-discussion context. At the same time, TOOL × EVAL was significant for both primary outcomes, and the difference between the AI-voice-assistant and smartphone-recording scenarios was larger under evaluation pressure. A cautious interpretation is that interactive preparation may be perceived as relatively more valuable when anticipated social-evaluative threat is higher. This interpretation is consistent with research on fear of negative evaluation, classroom environment, and online speaking anxiety that emphasises social visibility and evaluative risk (Horwitz et al., 1986; Khajavy et al., 2018; Bárkányi & Brash, 2025; Woodrow, 2006). This finding remains an interpretation derived from scenario-based responses.

The divergence between HWTC and speaking-initiation tendency in Study 2 also clarifies what the outcomes measure. For FLSA and HWTC, the AI–recording difference was larger under evaluation pressure; for speaking-initiation tendency, the AI–recording difference was larger without evaluation pressure, and the interaction was small (ηp^2^ = 0.008). In the case of speaking-initiation tendency, it focuses on the learners’ willingness to take their turn to speak, while in the case of HWTC, it combines readiness to answer, readiness to explain, readiness to ask, readiness to respond and readiness to summarise in the context of a human discussion. When it comes to publicity, there is no such thing as too soon. Grading allows students to wait or look on if they are not prepared to take part at this time but may wish to do so at a later stage. The two constructs are related yet cannot replace each other. The pressure of evaluation can be a greater limitation for early turn-taking than HWTC. This explanation is based upon a theoretical situation and one item regarding the order of speaking. It remains tentative. It would be useful to test it in the future by recording turn entry timing in real group discussions.

### 5.2. Theoretical Contributions

High-level research over the past three years has increasingly shifted from asking whether AI can be used for speaking learning to asking what kinds of AI interaction work, for whom, and under what conditions. Studies in the field show that different AI tools, degrees of embodiment, and interactional forms can produce different patterns in WTC, FLSA, and speaking outcomes (Fathi et al., 2024; C. Wang et al., 2024; W. Zhang, 2026; C. Zhang et al., 2024). Computer-Assisted Language Learning has begun to examine the integration of chatbots into speaking tasks, speech-enabled corrective feedback, interaction modes, and AI-based speaking assessment (Liu et al., 2025; Shadiev et al., 2026; Tai & Chen, 2025; T.-T. Wu et al., 2025). ReCALL has moved from meta-synthesis of speech-based chatbots toward AI-integrated speaking training and collaborative speaking learning (H. C. R. Chen et al., 2026; Koç & Savaş, 2025; T.-T. Wu et al., 2026). At the same time, theoretical discussions of GenAI and language education emphasise that the value of AI depends on how it is embedded in meaningful interpersonal, task, and instructional systems rather than simply on greater automation (Chapelle, 2024; González-Lloret, 2024; Handley, 2024; Kern, 2024; Thorne, 2024). This study builds on previous studies. It brings together the dichotomy between AI-driven and human-driven outcomes, the competence-belief–affect relationship and the evaluation context. The competence–affect–context account conceptualises human-directed communicative readiness following AI-assisted speaking practice. It is integrative and interpretive rather than one research design fully validated as one causal design.

This study is the first to connect AI-assisted speaking practice with learner engagement while maintaining clear construct boundaries. AISB indexes the self-reported intensity of AI-assisted speaking practice, whereas HWTC indexes human-directed communicative readiness toward teachers, peers, and less-familiar human interlocutors. FLSA is treated as an affective barrier, SSE as a competence belief and individual-difference condition, and evaluation pressure as an educational contextual condition. This mapping extends the analysis beyond AI interaction while preserving a clear evidentiary boundary. Human-directed communicative readiness and actual human communication are not equivalent to general cognitive or emotional engagement, speaking development, or transfer.

Second, the competence–affect component challenges the assumption that AI-assisted speaking practice works uniformly across all learners. FLSA formed a significant statistical indirect association in Study 1, while SSE altered the fitted FLSA–HWTC relationship. Johnson–Neyman and quadratic robustness analyses showed that the crossover was concentrated at the upper end of SSE and was not eliminated by simple curvature checks. This result makes a purely linear protective interpretation too coarse, but it does not show that anxiety becomes beneficial for highly self-efficacious learners or explain why the positive high-SSE region occurred. SSE should therefore be treated as an individual-difference boundary condition that requires replication with objective proficiency, repeated measures, and prospective or experimental data.

Third, Study 2 adds evaluation pressure as a contextual boundary of anticipated communicative engagement while also revealing an important design limitation. The AI–recording difference in anticipated FLSA and HWTC was larger under evaluation pressure, yet the AI × no-evaluation cell still showed the most favourable means. This pattern is consistent with the possibility that interactive rehearsal is perceived as especially useful when social-evaluative threat is high. The study also found that the preparation-tool manipulation was coupled with dialogue, responsiveness, and feedback, which combined with the AI identity could not be determined as the preparation mechanism alone or interactive preparation in general. A robust theoretical test would need to include a baseline condition that involves recording the dialogic speech pattern, as well as a matched dialogic speech pattern, using a human partner or a virtual partner with the capability to provide feedback.

### 5.3. Practical Implications

In terms of instruction, it is one viable model to have a sequence of increasing evaluative demands with independent preparation, then technology enhanced interactive rehearsal, then low risk human communication. Supportive digital tools can give follow-up questions, request for clarification, and feedback on vocabulary/expressions in the second stage. The third stage is an opportunity for learners to do the same or a similar topic through a peer group task, with no technology involved and interacting in real time. In the fourth stage, a gradual introduction of teachers’ grading and/or formal presentations demands can be made. Clearly structured tasks, dialogicality, social presence, collaborative arrangements and sustained interaction have been shown to affect the effectiveness of technology-supported speaking activities in prior research (Ebadi & Amini, 2024; Ericsson et al., 2023, 2024; Hsu et al., 2023; Hwang et al., 2024; Kim et al., 2022; T.-T. Wu et al., 2026). This sequence is a pedagogical proposal derived from the present findings and prior research, and it should be tested directly rather than treated as an established transfer pathway.

Task scaffolding may also be tailored to learners’ SSE and actual proficiency. This study does not confirm subgroup treatment effects. Future instructional trials for learners with lower SSE or weaker proficiency could test shorter speaking turns. They may add explicit lexical and sentence-frame support, plus repeated low-risk practice before learners interact with familiar peers. For learners with higher SSE or stronger proficiency, trials could remove scripted support faster. They are likely to introduce unexpected follow-up questions, counterarguments and real-time meaning negotiation. High self-efficacy does not mean learners need no human interaction. We did not measure objective proficiency, and the high-SSE crossover effect still lacks a full explanation. These suggestions remain instructional hypotheses instead of empirically validated guidelines.

Teachers may begin with ungraded or low-weight formative tasks that permit repeated attempts, peer feedback, and delayed teacher feedback and then increase grading weight and public-performance demands after learners have gained experience in human interaction. In Study 2, the AI–recording scenario difference was larger under formal evaluation for FLSA and HWTC, but the AI × no-evaluation scenario still showed the lowest FLSA and highest HWTC. This pattern should not be used as a reason to increase evaluative threat. Where high-stakes speaking assessment is unavoidable, AI can be considered as a rehearsal tool, while formal grades should rely primarily on human performance without AI and should avoid allowing AI-generated content to substitute for learners’ own organisation and expression. These recommendations require confirmation in actual classroom intervention studies.

Practice mode/assessment-preparation mode might be distinguished at the product level. In one mode, controllable follow-up questions, clarification requests, short corrections and re-expressions may be emphasised, while in another mode of operation, prompts may be gradually dispensed with, and time pressure, some unfamiliar questions and unscripted responses may be added in order to prepare for the assessment. Platforms could also track process data like how many practice turns were there, how many follow-up questions the learner came up with, how many times feedback was taken up, how many times the learner had to re-record, etc. This process data could help differentiate future studies between perceived support, actual process behaviour and human performance. Interfaces should not suggest that AI can replace valid evaluation anxiety; rather, it is rehearsal and scaffolding based on current evidence but not guaranteed transfer.

### 5.4. Limitations and Future Research

Study 1 adopted a cross-sectional self-report design and therefore cannot establish temporal order or causality. Common method bias and unmeasured confounding also remain possible. Although the ULMC sensitivity analysis reduced concerns about a dominant method factor, it could not fully eliminate common method bias. The contextualised short forms of FLSA, SSE, and HWTC showed acceptable reliability and discriminant validity, although the AVE for HWTC was borderline at 0.499. These adaptations were not independently evaluated through a formal expert-panel procedure or separate pilot testing. There was also a significant gender disparity in the sample and representation of participants ranged from diploma to doctoral level. Demographic-adjustment models were relatively stable, but there are limits to statistical adjustment for representativeness. AISB primarily observed the intensity with which a tool was used and did not differentiate between tool quality, task difficulty, feedback characteristics and learning strategies. Objective measures of speaking proficiency were not included in either study.

Study 2 had further methodological limitations. It was based on one-off scenarios to be imagined, and assignment was done via separate survey links instead of individual random allocation. Some perhaps insufficiently engaged responses might have been retained by the 45 s screening threshold and the manipulation checks might have been susceptible to demand characteristics. More importantly, the experimental comparison did not single out AI identity. The AI-voice-assistant condition bundled AI identity, dialogical interaction, and feedback, whereas the smartphone recording was monologic production without equivalent interaction. Accordingly, the observed between-condition differences cannot be attributed specifically to AI identity. Both studies were based on speech preparation without actual speech. The findings should therefore be interpreted as evidence concerning human-directed communicative readiness, rather than as evidence of actual behavioural transfer or improved speaking performance.

Future studies should help to better clarify the connection between AI-assisted speaking practice and human-directed communicative readiness, communicative behaviour and speaking performance. Platform logs and behavioural measures of speaking time, dialogue turns, response time and feedback received may be added to pre-task and post-task measures of FLSA, SSE and HWTC. Other internal dispositions should be separated from those of the task as well. Most importantly, future studies should include a common human-interaction task completed without concurrent digital assistance, so that speaking initiation, turn-taking, participation, and blind-rated performance can be directly assessed across conditions. Initiation of speech, turn-taking, and participation as well as blind-rated performance could then be measured. Follow-up activities would also be helpful in determining immediate readiness and sustained development if they were delayed.

The best way to further Study 2 would be to have a preregistered real-task random experiment, which would be more ecologically valid. Learners could be placed in similar preparation conditions: smartphone-recording condition, AI-voice-assistant condition, and human dialogic-partner condition. The preparation time, topic, amount of feedback and instructions of the task should be as similar as possible. The same human discussion task would then be completed by all groups. State FLSA, SSE, HWTC, speaking-initiation timing, participation, and blind-rated performance could be compared. Random assignment of the individual and multilevel modelling (in case of clustering at the classroom level) would enhance causal inference.

Replication of the SSE-related slope reversal is also needed. Further research is needed to determine if this crossover effect is maintained beyond differences in psychological characteristics and objective speaking proficiency. An examination of the pattern through multiple observations over a few speaking tasks and larger high-SSE samples would be useful in deciding whether the pattern is a stable boundary condition, a process connected to a particular subsample, or sampling instability. There is also a need for additional research to explore variations in AI systems, task types, and evaluative contexts. The timing of the translation of AI-assisted speaking practice to improved human-directed communicative readiness and human L2 communication may be influenced by response quality, corrective feedback, voice naturalness, AI literacy, real-life English use, disciplinary background, and cultural context.

## Figures and Tables

**Table 1:** Item-level standardised loadings and construct reliability/convergent validity for Study 1.

Construct	Standardised Item Loadings	CR	AVE	√AVE
FLSA	FLSA1 = 0.743; FLSA2 = 0.703; FLSA3 = 0.724; FLSA4 = 0.740; FLSA5 = 0.716	0.847	0.526	0.725
SSE	SSE1 = 0.708; SSE2 = 0.728; SSE3 = 0.704; SSE4 = 0.702; SSE5 = 0.698	0.834	0.502	0.708
HWTC	HWTC1 = 0.694; HWTC2 = 0.680; HWTC3 = 0.727; HWTC4 = 0.715; HWTC5 = 0.717	0.833	0.499	0.707

Note. CR = composite reliability; AVE = average variance extracted. HTMT values were FLSA–SSE = 0.491, FLSA–HWTC = 0.511, and SSE–HWTC = 0.485, all below the conservative 0.85 threshold, indicating adequate discriminant validity (Henseler et al., 2015). The square roots of AVE (0.725, 0.708, and 0.707, respectively) exceeded the corresponding absolute latent-factor correlations (0.489, 0.512, and 0.484), satisfying the Fornell–Larcker criterion. Although the AVE of HWTC was 0.499, marginally below the conventional 0.50 benchmark, the shortfall was only 0.001. Because AVE cutoffs are heuristic rather than formal statistical tests (Cheung et al., 2024), this value was interpreted together with HWTC’s satisfactory composite reliability (CR = 0.833), item loadings (0.680–0.727), and discriminant-validity evidence. Accordingly, convergent validity for HWTC was treated as borderline rather than unequivocally inadequate.

**Table 2:** Descriptive statistics and correlations for the core Study 1 variables.

Variable	M	SD	AISB	FLSA	SSE	HWTC
AISB	3.707	0.960	—	−0.416 **	0.368 **	0.384 **
FLSA	2.272	0.932		—	−0.413 **	−0.429 **
SSE	3.719	0.903			—	0.405 **
HWTC	3.746	0.898				—

Note. Core analyses were based on N = 708. ** *p* < 0.01.

**Table 3:** Main paths and conditional associations in Study 1.

Effect	Estimate	*p*/95% CI
AISB → HWTC (total association)	B = 0.359, β = 0.384	*p* < 0.001
AISB → FLSA	B = −0.404, β = −0.416	*p* < 0.001
FLSA → HWTC (controlling AISB)	B = −0.313, β = −0.325	*p* < 0.001
Indirect association	0.127	[0.079, 0.184]
FLSA × SSE	0.281	*p* < 0.001
Indirect association at low SSE	0.142	[0.097, 0.192]
Indirect association at mean SSE	0.044	[0.017, 0.077]
Indirect association at high SSE	−0.054	[−0.095, −0.013]

Note. The FLSA × SSE coefficient is from the simple moderation model used to test H3, whereas the conditional indirect associations are from the conditional-process model used to test H4. Indirect and conditional indirect confidence intervals are based on 5000 bootstrap resamples.

**Table 4:** Descriptive statistics for the main Study 2 variables by condition.

Variable	A: AI × Evaluation	B: AI × No Evaluation	C: Recording × Evaluation	D: Recording × No Evaluation
Perceived tool interactivity (MC)	3.90 (0.84)	3.90 (0.86)	2.50 (0.75)	2.50 (0.77)
Perceived evaluation pressure	3.90 (0.84)	2.50 (0.76)	3.90 (0.84)	2.50 (0.71)
FLSA	2.95 (0.67)	2.70 (0.82)	4.05 (0.94)	3.20 (0.82)
HWTC	3.55 (0.75)	3.60 (0.91)	2.50 (0.80)	3.30 (0.87)
Speaking-initiation tendency	3.25 (0.94)	3.86 (1.38)	2.95 (1.01)	3.14 (1.09)
Pre-scenario SSE	3.70 (0.92)	3.62 (0.92)	3.62 (0.95)	3.64 (0.99)
Scenario realism	3.30 (0.90)	3.25 (0.96)	3.27 (0.96)	3.15 (0.97)

Note. Valid sample sizes for Groups A/B/C/D were 167/188/184/187, respectively. Values in parentheses are SDs.

**Table 5:** Primary 2 × 2 ANOVA results and tool contrasts following significant interactions in Study 2.

Outcome	Effect or Contrast	Statistic/ΔM	*p*	Effect Size
FLSA	Preparation tool	F(1,722) = 171.07	<0.001	ηp^2^ = 0.192
FLSA	Evaluation pressure	F(1,722) = 81.74	<0.001	ηp^2^ = 0.102
FLSA	Tool × evaluation	F(1,722) = 23.73	<0.001	ηp^2^ = 0.032
FLSA	AI vs. recording under evaluation	ΔM = −1.10; t = −12.71	<0.001	d = −1.34
FLSA	AI vs. recording without evaluation	ΔM = −0.50; t = −5.89	<0.001	d = −0.61
HWTC	Preparation tool	F(1,722) = 116.97	<0.001	ηp^2^ = 0.139
HWTC	Evaluation pressure	F(1,722) = 46.56	<0.001	ηp^2^ = 0.061
HWTC	Tool × evaluation	F(1,722) = 35.59	<0.001	ηp^2^ = 0.047
HWTC	AI vs. recording under evaluation	ΔM = +1.05; t = 12.65	<0.001	d = 1.35
HWTC	AI vs. recording without evaluation	ΔM = +0.30; t = 3.28	0.001	d = 0.34
Speaking-initiation tendency	Preparation tool	F(1,722) = 37.75	<0.001	ηp^2^ = 0.050
Speaking-initiation tendency	Evaluation pressure	F(1,722) = 23.60	<0.001	ηp^2^ = 0.032
Speaking-initiation tendency	Tool × evaluation	F(1,722) = 6.11	0.014	ηp^2^ = 0.008
Speaking-initiation tendency	AI vs. recording under evaluation	t = 2.95	0.003	d = 0.31
Speaking-initiation tendency	AI vs. recording without evaluation	t = 5.59	<0.001	d = 0.58

Note. N = 726. ANOVA results use Type III sums of squares. For the tool contrasts, ΔM is AI minus recording. Tool contrasts were calculated from condition-specific *n*, M, and SD values using Welch’s t test and pooled-SD Cohen’s d. These estimates clarify the significant interactions but remain comparisons of anticipated responses under nonrandom scenario exposure.

**Table 6:** Demographic-adjusted ANCOVA summary controlling for SSE, prior AI use, gender, and education level.

Dependent Variable	Effect	df	F	*p*	ηp^2^
FLSA	SSE	1	64.75	<0.001	0.083
	Prior AI-use frequency	1	4.38	0.037	0.006
	Gender	1	0.68	0.411	0.001
	Education level	3	0.57	0.637	0.002
	Preparation tool	1	204.90	<0.001	0.223
	Evaluation pressure	1	95.26	<0.001	0.117
	Preparation tool × Evaluation pressure	1	31.30	<0.001	0.042
HWTC	SSE	1	72.60	<0.001	0.092
	Prior AI-use frequency	1	4.18	0.041	0.006
	Gender	1	4.06	0.044	0.006
	Education level	3	1.42	0.235	0.006
	Preparation tool	1	140.95	<0.001	0.164
	Evaluation pressure	1	57.47	<0.001	0.074
	Preparation tool × Evaluation pressure	1	39.01	<0.001	0.052

Note. N = 726. Type III sums of squares were used. Education level was treated as a categorical covariate. Residual df = 716; effect df = 1 except education level (df = 3). ηp^2^ denotes partial eta squared.

## Data Availability

The data that support the findings of this study are available on request from the corresponding author. The data are not publicly available due to privacy or ethical restrictions.

## Appendix A. Measure Sources, Scoring, and Exact Administered Items

### Appendix A.1. Study 1

AISB coding. Questions 7–9 referred to the preceding four weeks. The ordered category codes were averaged with equal weights: AISB = (session code + cumulative-time code + use-days code)/3.

Sessions. 0 sessions = 0; 1–2 = 1; 3–5 = 2; 6–9 = 3; 10–19 = 4; 20 or more = 5.

Minutes. 0 min = 0; 1–15 = 1; 16–30 = 2; 31–60 = 3; 61–120 = 4; 121 or more = 5.

Days. 0 days = 0; 1–3 = 1; 4–7 = 2; 8–12 = 3; 13–20 = 4; 21–28 = 5.

FLSA (5 items). Selected and contextually adapted from Woodrow (2006). Response scale: 1 = Strongly disagree, 2 = Disagree, 3 = Neither agree nor disagree, 4 = Agree, 5 = Strongly agree. The scale score was the mean of the five items.

1. I feel nervous when I have to respond in English without enough preparation.
2. I worry about making mistakes when speaking English.
3. I feel self-conscious when other people are listening to me speak English.
4. I feel anxious when speaking English with people who are more proficient than I am.
5. When I feel nervous, I often cannot recall English words or expressions that I normally know.

SSE (5 items). Developed with reference to Gan et al. (2022) and Y. Wang and Sun (2024). Response scale: 1 = Cannot do at all, 2 = Can do only with difficulty, 3 = Can do to some extent, 4 = Can do well, 5 = Can do very well. The scale score was the mean of the five items.

1. I can express my ideas clearly in English.
2. I can organize my spoken English logically and coherently.
3. When speaking English, I can choose appropriate vocabulary and grammatical structures.
4. I can pronounce English clearly enough for listeners to understand me.
5. Even when I have difficulty expressing myself, I can find ways to keep an English conversation going.

HWTC (5 items). Selected and contextually adapted from human communication situations in J. S. Lee et al. (2021). Response scale: 1 = Definitely unwilling, 2 = Probably unwilling, 3 = Not sure, 4 = Probably willing, 5 = Definitely willing. The scale score was the mean of the five items.

1. I would be willing to answer a teacher’s or lecturer’s question in English.
2. I would be willing to ask a teacher or lecturer a question in English.
3. I would be willing to have a one-to-one conversation in English with a classmate or friend.
4. I would be willing to join an English discussion with several people.
5. I would be willing to start a conversation in English with someone I do not know well.

### Appendix A.2. Study 2

Pre-scenario SSE (5 items). Stem: “When I need to communicate in English with real people, I can…” Response scale: 1 = Cannot do at all, 2 = Can do with great difficulty, 3 = Can basically do, 4 = Can do fairly well, 5 = Can do completely.

1. Express my ideas clearly.
2. Organize my spoken ideas logically and coherently.
3. Choose appropriate vocabulary and grammar to express my meaning.
4. Pronounce words clearly enough to be understood.
5. Keep the conversation going when I have difficulty expressing myself.

Scenario-specific FLSA (5 items). Response scale: 1 = Strongly disagree, 2 = Disagree, 3 = Neither agree nor disagree, 4 = Agree, 5 = Strongly agree.

1. I would feel nervous if I had to speak first in the group.
2. I would worry about making mistakes in English in front of the other students.
3. I would feel uncomfortable when the other students pay attention to my English.
4. I would feel anxious if the other students were more proficient in English than I am.
5. Nervousness would make it difficult for me to find the right words while speaking.

Scenario-specific HWTC (5 items). Stem: “In the real group-discussion scenario, how willing would you be to do each of the following?” Response scale: 1 = Definitely unwilling, 2 = Probably unwilling, 3 = Unsure, 4 = Probably willing, 5 = Definitely willing.

1. Volunteer to speak first.
2. Explain my view and reasons clearly to the group.
3. Ask another student a follow-up question.
4. Respond to a student whose opinion differs from mine.
5. Volunteer to summarize the group’s main ideas.

Speaking-order item. Question: “If you could choose when to speak, which option would you most likely choose?” Options: Speak first; Speak second; Speak after listening to the others; Speak only if called on; Try not to speak. The manuscript reverse-scored this item as the secondary speaking-initiation tendency indicator.

Manipulation checks and scenario realism. Response scale for all six statements: 1 = Strongly disagree, 2 = Disagree, 3 = Neither agree nor disagree, 4 = Agree, 5 = Strongly agree.

1. MC1. The preparation tool could respond to my spoken English and continue a dialogue with me.
2. MC2. The preparation tool could give immediate feedback on my spoken English.
3. EVAL1. The English teacher would formally evaluate my individual spoken-English performance.
4. EVAL2. My performance in the discussion would count toward my course grade.
5. REAL1. I could clearly imagine the situation that was described.
6. REAL2. The situation described is realistic and believable for university students.

Prior AI voice-use frequency. Question: “During the past four weeks, about how often did you use an AI voice feature for spoken English conversation?” Options: Never; Less than once a month; 1–3 times a month; 1–2 times a week; 3 or more times a week.

## References

[B1-behavsci-16-01770] Annamalai N., Ab Rashid R., Hashmi U. M., Mohamed M., Alqaryouti M. H., Sadeq A. E. (2023). Using chatbots for English language learning in higher education. Computers and Education: Artificial Intelligence.

[B2-behavsci-16-01770] Ballıdağ M., Aydın S. (2025). A comparison of the effects of AI-based chatbots and peer interactions on speaking anxiety among EFL learners. Future in Educational Research.

[B3-behavsci-16-01770] Bandura A. (1997). Self-efficacy: The exercise of control.

[B4-behavsci-16-01770] Bárkányi Z. (2021). Motivation, self-efficacy beliefs, and speaking anxiety in language MOOCs. ReCALL.

[B5-behavsci-16-01770] Bárkányi Z., Brash B. (2025). Foreign language speaking anxiety online: Mitigating strategies and speaking practices. ReCALL.

[B6-behavsci-16-01770] Botes E., Dewaele J.-M., Greiff S. (2020). The foreign language classroom anxiety scale and academic achievement: An overview of the prevailing literature and a meta-analysis. Journal for the Psychology of Language Learning.

[B7-behavsci-16-01770] Caldarini G., Jaf S., McGarry K. (2022). A literature survey of recent advances in chatbots. Information.

[B8-behavsci-16-01770] Cao Y. (2011). Investigating situational willingness to communicate within second language classrooms from an ecological perspective. System.

[B9-behavsci-16-01770] Chapelle C. A. (2024). Open generative AI changes a lot, but not everything. The Modern Language Journal.

[B10-behavsci-16-01770] Chen H. C. R., Zhou X., Tian J. X. (2026). Examining the effectiveness of integrating corpus-based and AI approaches for English speaking practice. ReCALL.

[B11-behavsci-16-01770] Chen H. H.-J., Yang C. T.-Y., Lai K. K.-W. (2023). Investigating college EFL learners’ perceptions toward the use of Google Assistant for foreign language learning. Interactive Learning Environments.

[B12-behavsci-16-01770] Chen Y.-C. (2024). Effects of technology-enhanced language learning on reducing EFL learners’ public speaking anxiety. Computer Assisted Language Learning.

[B13-behavsci-16-01770] Cheung G. W., Cooper-Thomas H. D., Lau R. S., Wang L. C. (2024). Reporting reliability, convergent and discriminant validity with structural equation modeling: A review and best-practice recommendations. Asia Pacific Journal of Management.

[B14-behavsci-16-01770] Çakmak F. (2022). Chatbot-human interaction and its effects on EFL students’ L2 speaking performance and anxiety. Novitas-ROYAL (Research on Youth and Language).

[B15-behavsci-16-01770] Davin K. J. (2024). The issue: New technologies and language education. The Modern Language Journal.

[B16-behavsci-16-01770] Dizon G. (2017). Using intelligent personal assistants for second language learning: A case study of Alexa. TESOL Journal.

[B17-behavsci-16-01770] Dizon G. (2020). Evaluating intelligent personal assistants for L2 listening and speaking development. Language Learning & Technology.

[B18-behavsci-16-01770] Dizon G., Tang D. (2020). Intelligent personal assistants for autonomous second language learning: An investigation of Alexa. The JALT CALL Journal.

[B19-behavsci-16-01770] Du J., Daniel B. K. (2024). Transforming language education: A systematic review of AI-powered chatbots for English as a foreign language speaking practice. Computers and Education: Artificial Intelligence.

[B20-behavsci-16-01770] Du Y., Reynolds B. L. (2025). Rooted in and beyond interaction: A systematic review of interactive affordances of chatbots for language learning amidst the rise of large language models. Acta Psychologica.

[B21-behavsci-16-01770] Ebadi S., Amini A. (2024). Examining the roles of social presence and human-likeness on Iranian EFL learners’ motivation using artificial intelligence technology: A case of CSIEC chatbot. Interactive Learning Environments.

[B22-behavsci-16-01770] Ebadi S., Azizimajd H. (2026). Investigating the effects of speaking tasks in Clubhouse on EFL learners’ speaking and foreign language speaking anxiety. Computer Assisted Language Learning.

[B23-behavsci-16-01770] El Shazly R. (2021). Effects of artificial intelligence on English speaking anxiety and speaking performance: A case study. Expert Systems.

[B24-behavsci-16-01770] Ericsson E., Lundin J., Sofkova Hashemi S. (2024). From deadpan machine to relating socially: Middle school students’ experiences speaking English with embodied conversational agents. Journal of Research on Technology in Education.

[B25-behavsci-16-01770] Ericsson E., Sofkova Hashemi S., Lundin J. (2023). Fun and frustrating: Students’ perspectives on practicing speaking English with virtual humans. Cogent Education.

[B26-behavsci-16-01770] Fathi J., Rahimi M., Derakhshan A. (2024). Improving EFL learners’ speaking skills and willingness to communicate via artificial intelligence-mediated interactions. System.

[B27-behavsci-16-01770] Fredricks J. A., Blumenfeld P. C., Paris A. H. (2004). School engagement: Potential of the concept, state of the evidence. Review of Educational Research.

[B28-behavsci-16-01770] Freiermuth M., Jarrell D. (2006). Willingness to communicate: Can online chat help?. International Journal of Applied Linguistics.

[B29-behavsci-16-01770] Fryer L. K., Ainley M., Thompson A., Gibson A., Sherlock Z. (2017). Stimulating and sustaining interest in a language course: An experimental comparison of chatbot and human task partners. Computers in Human Behavior.

[B30-behavsci-16-01770] Fryer L. K., Nakao K., Thompson A. (2019). Chatbot learning partners: Connecting learning experiences, interest and competence. Computers in Human Behavior.

[B31-behavsci-16-01770] Fryer L. K., Thompson A., Nakao K., Howarth M., Gallacher A. (2020). Supporting self-efficacy beliefs and interest as educational inputs and outcomes: Framing AI and human partnered task experiences. Learning and Individual Differences.

[B32-behavsci-16-01770] Gan Z., Yan Z., An Z. (2022). Development and validation of an EFL speaking self-efficacy scale in the self-regulated learning context. The Journal of Asia TEFL.

[B33-behavsci-16-01770] Goh C. C. M., Aryadoust V. (2025). Developing and assessing second language listening and speaking: Does AI make it better?. Annual Review of Applied Linguistics.

[B34-behavsci-16-01770] González-Lloret M. (2024). The future of language learning and teaching in a technology-mediated 21st century. The Modern Language Journal.

[B35-behavsci-16-01770] Han J., Lee D. (2024). Research on the development of principles for designing elementary English speaking lessons using artificial intelligence chatbots. Humanities and Social Sciences Communications.

[B36-behavsci-16-01770] Handley Z. (2024). Has artificial intelligence rendered language teaching obsolete?. The Modern Language Journal.

[B37-behavsci-16-01770] Henseler J., Ringle C. M., Sarstedt M. (2015). A new criterion for assessing discriminant validity in variance-based structural equation modeling. Journal of the Academy of Marketing Science.

[B38-behavsci-16-01770] Horwitz E. K., Horwitz M. B., Cope J. (1986). Foreign language classroom anxiety. The Modern Language Journal.

[B39-behavsci-16-01770] Hsu M.-H., Chen P.-S., Yu C.-S. (2023). Proposing a task-oriented chatbot system for EFL learners speaking practice. Interactive Learning Environments.

[B40-behavsci-16-01770] Huang W., Hew K. F., Fryer L. K. (2022). Chatbots for language learning—Are they really useful? A systematic review of chatbot-supported language learning. Journal of Computer Assisted Learning.

[B41-behavsci-16-01770] Hwang W.-Y., Guo B.-C., Hoang A., Chang C.-C., Wu N.-T. (2024). Facilitating authentic contextual EFL speaking and conversation with smart mechanisms and investigating its influence on learning achievements. Computer Assisted Language Learning.

[B42-behavsci-16-01770] Jeon J. (2024). Exploring AI chatbot affordances in the EFL classroom: Young learners’ experiences and perspectives. Computer Assisted Language Learning.

[B43-behavsci-16-01770] Jeon J., Lee S., Choe H. (2023). Beyond ChatGPT: A conceptual framework and systematic review of speech-recognition chatbots for language learning. Computers & Education.

[B44-behavsci-16-01770] Jeon J., Lee S., Choi S. (2024). A systematic review of research on speech-recognition chatbots for language learning: Implications for future directions in the era of large language models. Interactive Learning Environments.

[B45-behavsci-16-01770] Jia H., Han I., Ko Y. (2023). A systematic review of conversational AI in language education: Focusing on the collaboration with human teachers. Journal of Research on Technology in Education.

[B46-behavsci-16-01770] Kern R. (2024). Twenty-first century technologies and language education: Charting a path forward. The Modern Language Journal.

[B47-behavsci-16-01770] Khajavy G. H., MacIntyre P. D., Barabadi E. (2018). Role of the emotions and classroom environment in willingness to communicate: Applying doubly latent multilevel analysis in second language acquisition research. Studies in Second Language Acquisition.

[B48-behavsci-16-01770] Kim H., Yang H., Shin D., Lee J. H. (2022). Design principles and architecture of a second language learning chatbot. Language Learning & Technology.

[B49-behavsci-16-01770] Koç F. Ş., Savaş P. (2025). The use of artificially intelligent chatbots in English language learning: A systematic meta-synthesis study of articles published between 2010 and 2024. ReCALL.

[B50-behavsci-16-01770] Kohnke L., Moorhouse B. L., Zou D. (2023). ChatGPT for language teaching and learning. RELC Journal.

[B51-behavsci-16-01770] Lai W. Y. W., Lee J. S. (2024). A systematic review of conversational AI tools in ELT: Publication trends, tools, research methods, learning outcomes, and antecedents. Computers and Education: Artificial Intelligence.

[B52-behavsci-16-01770] Lee J. S., Sylvén L. K., Lee K. (2021). Cross-cultural insights into Korean and Swedish secondary school students’ willingness to communicate in a second language. Journal of Multilingual and Multicultural Development.

[B53-behavsci-16-01770] Lee K.-A., Lim S.-B. (2023). Designing a leveled conversational teachable agent for English language learners. Applied Sciences.

[B54-behavsci-16-01770] Lee S., Jeon J. (2024). Visualizing a disembodied agent: Young EFL learners’ perceptions of voice-controlled conversational agents as language partners. Computer Assisted Language Learning.

[B55-behavsci-16-01770] Li C., Dewaele J.-M., Pawlak M., Kruk M. (2025). Classroom environment and willingness to communicate in English: The mediating role of emotions experienced by university students in China. Language Teaching Research.

[B56-behavsci-16-01770] Li Z., Li B., Wang X., Zhen L. (2025). Clustering perceived learning environment as antecedents of willingness to communicate in a second language. Language Teaching Research.

[B57-behavsci-16-01770] Liu X., Huang J., Deng Y., Spiridakis J. (2025). AI versus human assessment in EFL speaking classrooms: A comparative study in China. Computer Assisted Language Learning.

[B58-behavsci-16-01770] MacIntyre P. D. (2007). Willingness to communicate in the second language: Understanding the decision to speak as a volitional process. The Modern Language Journal.

[B59-behavsci-16-01770] MacIntyre P. D., Clément R., Dörnyei Z., Noels K. A. (1998). Conceptualizing willingness to communicate in a L2: A situational model of L2 confidence and affiliation. The Modern Language Journal.

[B60-behavsci-16-01770] Mills N., Pajares F., Herron C. (2007). Self-efficacy of college intermediate French students: Relation to achievement and motivation. Language Learning.

[B61-behavsci-16-01770] Moussalli S., Cardoso W. (2020). Intelligent personal assistants: Can they understand and be understood by accented L2 learners?. Computer Assisted Language Learning.

[B62-behavsci-16-01770] Peng J.-E., Liang W. (2025). Willingness to communicate with artificial intelligence (AI)? Insights from tracking EFL learners’ perceived acceptance and chat output. Computer Assisted Language Learning.

[B63-behavsci-16-01770] Peng J.-E., Woodrow L. (2010). Willingness to communicate in English: A model in the Chinese EFL classroom context. Language Learning.

[B64-behavsci-16-01770] Shadiev R., Feng Y., Zhussupova R., Altinay F. (2026). Effects of speech-enabled corrective feedback technology on EFL speaking skills, anxiety and confidence. Computer Assisted Language Learning.

[B65-behavsci-16-01770] Sok S., Shin H. W. (2025). Do interactions with ChatGPT influence L2 learners’ oral speaking ability, summarization ability, and perceptions of generative AI tasks?. TESOL Quarterly.

[B66-behavsci-16-01770] Tai T.-Y. (2026). ChatGPT-assisted EFL learning beyond the classroom: Impact on oral proficiency, enjoyment, and grit. Computer Assisted Language Learning.

[B67-behavsci-16-01770] Tai T.-Y., Chen H. H.-J. (2025). Impact of generative AI chatbots and interaction modes on the speaking proficiency of adolescent EFL learners. Computer Assisted Language Learning.

[B68-behavsci-16-01770] Thorne S. L. (2024). Generative artificial intelligence, co-evolution, and language education. The Modern Language Journal.

[B69-behavsci-16-01770] Topalov J., Knežević L., Halupka-Rešetar S. (2023). How anxious are online ESP learners? Exploring students’ anxiety in video, audio and text-based communication in an online classroom. ESP Today.

[B70-behavsci-16-01770] Tutton M., Cohen D. L. (2024). Foreign language anxiety and self-disclosure in online university French classes. Language Teaching Research.

[B71-behavsci-16-01770] Wang C., Zou B., Du Y., Wang Z. (2024). The impact of different conversational generative AI chatbots on EFL learners: An analysis of willingness to communicate, foreign language speaking anxiety, and self-perceived communicative competence. System.

[B72-behavsci-16-01770] Wang X., Liu Q., Pang H., Tan S. C., Lei J., Wallace M. P., Li L. (2023). What matters in AI-supported learning: A study of human-AI interactions in language learning using cluster analysis and epistemic network analysis. Computers & Education.

[B73-behavsci-16-01770] Wang Y., Sun P. P. (2024). Development and validation of scales for speaking self-efficacy: Constructs, sources, and relations. PLoS ONE.

[B74-behavsci-16-01770] Woodrow L. (2006). Anxiety and speaking English as a second language. RELC Journal.

[B75-behavsci-16-01770] Wu C. H., Lam H. Y., Kong A., Wong W. L. H. (2023). The design and evaluation of a digital learning-based English chatbot as an online self-learning method. International Journal of Engineering Business Management.

[B76-behavsci-16-01770] Wu T.-T., Hapsari I. P., Huang Y.-M. (2025). Effects of incorporating AI chatbots into think–pair–share activities on EFL speaking anxiety, language enjoyment, and speaking performance. Computer Assisted Language Learning.

[B77-behavsci-16-01770] Wu T.-T., Hapsari I. P., Huang Y.-M. (2026). Intelligent chatbot–supported collaborative learning: Impact on student engagement and English speaking skills. ReCALL.

[B78-behavsci-16-01770] Xiao F., Zhao P., Sha H., Yang D., Warschauer M. (2024). Conversational agents in language learning. Journal of China Computer-Assisted Language Learning.

[B79-behavsci-16-01770] Yang C. T.-Y., Lai S.-L., Chen H. H.-J. (2024). The impact of intelligent personal assistants on learners’ autonomous learning of second language listening and speaking. Interactive Learning Environments.

[B80-behavsci-16-01770] Yashima T. (2002). Willingness to communicate in a second language: The Japanese EFL context. The Modern Language Journal.

[B81-behavsci-16-01770] Yuan Y. (2024). An empirical study of the efficacy of AI chatbots for English as a foreign language learning in primary education. Interactive Learning Environments.

[B82-behavsci-16-01770] Zhai C., Wibowo S. (2022). A systematic review on cross-culture, humor and empathy dimensions in conversational chatbots: The case of second language acquisition. Heliyon.

[B83-behavsci-16-01770] Zhang C., Meng Y., Ma X. (2024). Artificial intelligence in EFL speaking: Impact on enjoyment, anxiety, and willingness to communicate. System.

[B84-behavsci-16-01770] Zhang W. (2026). The impact of AI chatbots on EFL learners’ oral proficiency and willingness to communicate. System.

[B85-behavsci-16-01770] Zou B., Wang C., Yan Y., Du X., Ji Y. (2025). Exploring English as a Foreign Language learners’ adoption and utilisation of ChatGPT for speaking practice through an extended technology acceptance model. International Journal of Applied Linguistics.

